# Dopamine, psychosis and schizophrenia: the widening gap between basic and clinical neuroscience

**DOI:** 10.1038/s41398-017-0071-9

**Published:** 2018-01-31

**Authors:** JP Kesby, DW Eyles, JJ McGrath, JG Scott

**Affiliations:** 1 Queensland Brain Institute, The University of Queensland, St. Lucia, QLD Australia; 2 Centre for Clinical Research, Faculty of Medicine, The University of Queensland, Herston, QLD Australia; 3 Queensland Centre for Mental Health Research, The Park Centre for Mental Health, Wacol, QLD Australia; 4 National Centre for Register-based Research, Aarhus University, Aarhus C, Denmark; 5 Metro North Mental Health, Royal Brisbane and Women’s Hospital, Herston, QLD Australia

## Abstract

The stagnation in drug development for schizophrenia highlights the need for better translation between basic and clinical research. Understanding the neurobiology of schizophrenia presents substantial challenges but a key feature continues to be the involvement of subcortical dopaminergic dysfunction in those with psychotic symptoms. Our contemporary knowledge regarding dopamine dysfunction has clarified where and when dopaminergic alterations may present in schizophrenia. For example, clinical studies have shown patients with schizophrenia show increased presynaptic dopamine function in the associative striatum, rather than the limbic striatum as previously presumed. Furthermore, subjects deemed at high risk of developing schizophrenia show similar presynaptic dopamine abnormalities in the associative striatum. Thus, our view of subcortical dopamine function in schizophrenia continues to evolve as we accommodate this newly acquired information. However, basic research in animal models has been slow to incorporate these clinical findings. For example, psychostimulant-induced locomotion, the commonly utilised phenotype for positive symptoms in rodents, is heavily associated with dopaminergic activation in the limbic striatum. This anatomical misalignment has brought into question how we assess positive symptoms in animal models and represents an opportunity for improved translation between basic and clinical research. The current review focuses on the role of subcortical dopamine dysfunction in psychosis and schizophrenia. We present and discuss alternative phenotypes that may provide a more translational approach to assess the neurobiology of positive symptoms in schizophrenia. Incorporation of recent clinical findings is essential if we are to develop meaningful translational animal models.

## Introduction

Our knowledge of the neurobiology of schizophrenia, while still rudimentary, has advanced considerably in recent years. However, these findings have not translated to better treatments for those with schizophrenia. The three primary symptom groups, positive, cognitive and negative (Box 1), have been associated with reports of abnormalities in virtually every neurotransmitter system^1–5^. The onset of psychotic symptoms, which is strongly associated with alterations in dopamine function, is a key feature underpinning a clinical diagnosis^6, 7^. However, results from clinical research regarding the specific loci of dopamine dysfunction in schizophrenia^8–10^, have triggered a reappraisal of our perspective on the neurobiology of schizophrenia. Currently there is a disparity between the tests for positive symptoms in animal models and recent clinical evidence for dopaminergic abnormalities in schizophrenia. Therefore, it is critical that this contemporary clinical knowledge actively influences the agenda in applied basic neuroscience.

### Box 1: Symptom groups in schizophrenia

- *Positive symptoms*
  *:* Positive symptoms include delusions and hallucinations, linked to aberrant salience. These symptoms are most recognisable during periods of acute psychosis.
- *Cognitive symptoms*
  *:* Impairments in learning, memory, attention and executive functioning are all included as cognitive symptoms.
- *Negative symptoms:* Negative symptoms include blunting of affect (lacking emotional expression), avolition (deficits in motivation) and social withdrawal.

It is widely acknowledged that we cannot recreate the complicated symptom profile of schizophrenia in animal models. However, animal models (the majority and focus of the present article being rodent models) provide an avenue to invasively explore the role of neurotransmitters and circuitry in psychiatric diseases. To improve the poor predictive validity of treatments in animal models^11^, it is critical that our understanding and the use of animal models evolves alongside our knowledge of schizophrenia neurobiology. The delayed incorporation of new clinical findings to develop better animal models highlights the need for better communication between clinical and basic research communities.

In this article, we discuss the challenges clinicians and researchers are facing in understanding the neurobiology of positive symptoms and psychosis in schizophrenia. We discuss the implications this has for current assessments of positive symptoms in rodents and propose a more relevant set of tests for future study. Finally, the need for a joint focus on bi-directional translation between clinical and basic research is outlined.

## Challenges in diagnosing schizophrenia

Psychiatric symptoms exist on continua from normal to pathological, meaning the threshold for diagnosis of schizophrenia in clinical practice can be challenging. The clinical diagnosis of schizophrenia relies heavily on the positive symptoms associated with a prolonged psychotic episode. However, a relatively high percentage of the general population (8–30%) report delusional experiences or hallucinations in their lifetime^12–14^, but for most people these are transient^15^. Psychotic symptoms are also not specific to a particular mental disorder^16^. The clinical efficacy of antipsychotic drugs is heavily correlated with their ability to block subcortical dopamine D2 receptors^17, 18^, suggesting dopamine signalling is important. In spite of this, no consistent relationship between D2 receptors and the pathophysiology of schizophrenia has emerged^19, 20^. In contrast, the clinical evidence points towards presynaptic dopamine dysfunction as a mediator of psychosis in schizophrenia^19^.

## The neurobiology of psychosis: the centrality of dopamine

### Dopamine systems: anatomy and function

**Fig. 1:**
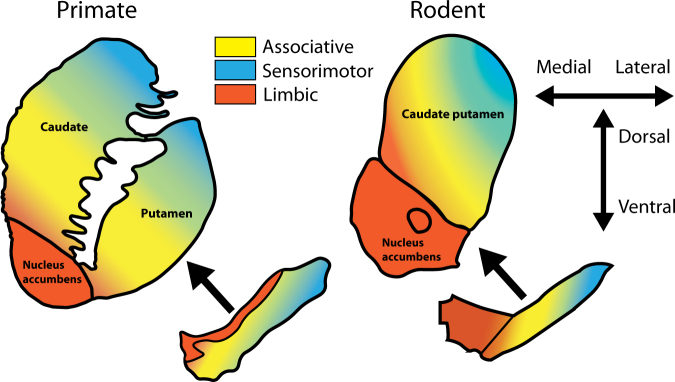
Functional subdivisions of the dopamine system across species. Midbrain dopamine neurons are the source of dopamine projections to the striatum in primates (left) and rodents (right). Important neuroanatomical differences exist, especially when considering functional subdivisions of the striatum. In the primate, the limbic system (orange) originates in the dorsal tier of the substantia nigra (the ventral tegmental area equivalent). In the rodent, the limbic system originates in ventral tegmental area, which sits medially to the substantia nigra. The midbrain projections to the associative striatum (yellow) and sensorimotor striatum (blue) follow a dorsomedial-to-ventrolateral topology

An appreciation for the neuroanatomical differences in subcortical dopaminergic projections/circuitry between rodents and primates is essential for effective communication between clinical and basic researchers. For example, primates feature a more prominent substantia nigra and less distinctive ventral tegmental area than rodents. However, more pertinent to the current review are homologous functional subdivisions of the striatum observed in both rodents and primates^21–24^. These include the limbic, associative and sensorimotor areas (Fig. 1). The associative striatum, defined by its dense connectivity from the frontal and parietal associative cortices, is key for goal-directed action and behavioural flexibility. The limbic striatum, defined by connectivity to the hippocampus, amygdala and medial orbitofrontal cortex, is involved in reward and motivation. The sensorimotor striatum, defined by connectivity to sensory and motor cortices, is critical for habit formation. These functional subdivisions are also interconnected by feedforward striato-nigro-striatal projections^25^. The heavy basis on behavioural outcomes in neuropsychiatry has made functional subdivisions such as these more relevant than ever.

### Dopaminergic features of psychosis in schizophrenia

In healthy individuals, dopamine stimulants such as amphetamine can induce psychotic symptoms^26, 27^ and people with schizophrenia are more sensitive to these effects^27, 28^. Studies using positron emission tomography (PET) imaging have shown patients with schizophrenia show increases in subcortical synaptic dopamine content^29, 30^, abnormally high dopamine release after amphetamine treatment^30–35^ and increased basal dopamine synthesis capacity (determined indirectly by increased radiolabelled L-DOPA uptake)^19,36, 37^ compared with healthy controls. Increased subcortical dopamine synthesis and release capacity are strongly associated with positive symptoms in patients^33, 38^, and increased subcortical synaptic dopamine content is predictive of a positive treatment response^29^. It was widely anticipated that the limbic striatum would be confirmed as the subdivision where these alterations in dopamine function would be localised in patients. The basis for this prediction was the belief that reward systems were aberrant in schizophrenia^39^. However, as PET imaging resolution improved it was found that increases in synaptic dopamine content^9, 10^ and synthesis capacity^8^ were localised, or more pronounced^37^, in the associative striatum (Fig. 1; yellow). Furthermore, alterations in dopamine function within the associative striatum likely contribute to the misappropriate attribution of salience to certain stimuli, a key aspect of delusions and psychosis^40^.

Clinical studies have confirmed that dopamine abnormalities are also present prior to the onset of psychosis in schizophrenia and thus are not a consequence of psychotic episodes or antipsychotic exposure. Similar to what has been observed in patients with schizophrenia, ultra-high risk (UHR) subjects show increased subcortical synaptic dopamine content^41^ and basal dopamine synthesis capacity^8, 42–44^. Importantly, alterations in dopamine synthesis capacity in UHR subjects progress over time^45^ and are greater in subjects who transition to psychosis compared with those who do not^46^. Furthermore, higher baseline synaptic dopamine levels in UHR subjects predicts a greater reduction in positive symptoms after dopamine depletion^41^. Overall, these findings in UHR subjects are congruent with those observed in schizophrenia and provide evidence indicating that presynaptic dopaminergic abnormalities are present prior to the onset of psychosis.

Several avenues have been proposed to explain a selective increase in associative striatal dopamine function, such as alterations in hippocampal control of dopamine projections^47, 48^, alterations in cortical inputs to midbrain dopamine systems^2, 49^ and, although little direct evidence has been observed, developmental alterations in dopamine neurons themselves^50, 51^. Furthermore, other pathways and/or neurotransmitters may be more critical in treatment-resistant patients^52^. We propose a network model whereby dysfunction in a central circuit, including the associative striatum, prefrontal cortex and thalamus, is critical for the expression of psychotic symptoms in schizophrenia. This model would suggest that dysfunction in auxiliary circuits (both limbic and cortical) contribute to psychotic symptoms by feeding into this primary network. Ascertaining the role of dopaminergic dysfunction, in the context of networks important for psychotic symptoms in schizophrenia, will provide a better base for constructing objective readouts in basic and clinical research.

## Psychosis: a consequence of network dysfunction

Psychosis is a condition that features a range of behavioural alterations that relate to a loss of contact with reality and a loss of insight. People with psychosis experience hallucinations (primarily auditory in schizophrenia^53^) and delusions. In schizophrenia, auditory hallucinations have been associated with altered connectivity between the hippocampus and thalamus^54^. During hallucinations, increased activation of the thalamus, striatum and hippocampus have also been observed^55^. Thus, altered thalamocortical connectivity, especially with the hippocampus, may impede internal/external representations of auditory processing^56^. In contrast, delusions in people with schizophrenia have been associated with overactivation of the prefrontal cortex (PFC) and diminished deactivation of striatal and thalamic networks^57^. Thus, the complexity of psychotic symptoms is congruent with the highly connected nature of implicated brain regions.

Although we still know little about the underlying neurobiology of psychosis, focal brain lesions allow for a better understanding of the networks involved without the confounds of medication and unrelated neuropathology. Generally speaking, lesions that induce hallucinations are often in the brain networks associated with the stimulus of the hallucination (i.e., auditory, visual or somatosensory)^58^. Visual hallucinations have been associated with dysfunction of the occipital lobe, striatum and thalamus, whereas auditory hallucinations are associated with dysfunction of the temporal lobe, hippocampus, amygdala and thalamus^58^. Insight is generally maintained after focal brain lesions that produce hallucinations and subcortical dopamine function is normal^59^, unlike what is observed in schizophrenia^58^. In contrast, a loss of insight (which can manifest as delusionary beliefs) is associated with alterations in cortico-striatal networks. For example, people with basal ganglia or caudate lesions can present with both hallucinations and delusions^60, 61^. Furthermore, a case study of religious delusions in a patient with temporal lobe epilepsy was associated with overactivity of the PFC^62^, and there are multiple lines of evidence suggesting that the PFC is integral for delusionary beliefs^63^. Therefore, while impairing networks specific to certain sensory modalities can lead to hallucinations, dysfunctional integration of PFC input to the associative striatum may be especially important for delusional symptoms in schizophrenia.

**Fig. 2:**
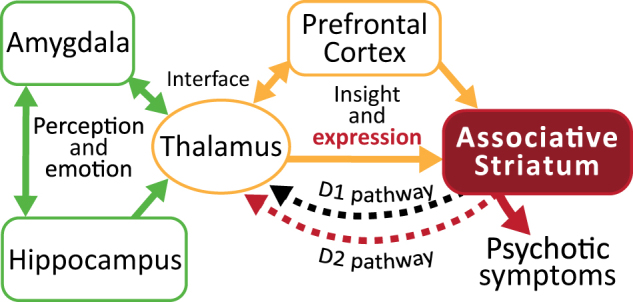
Network implicated in psychotic symptoms and schizophrenia. Dysfunction in a variety of brain regions can elicit psychotic symptoms. A primary circuit involved in psychosis includes the thalamus and prefrontal cortex (yellow) feeding into the associative striatum. Alterations in the thalamus and prefrontal cortex are involved in hallucinations and also insight for delusional symptoms. Expression of psychotic symptoms in most cases requires increased activity in the associative striatum and specifically excessive D2 receptor stimulation (red). Other limbic regions such as the hippocampus and amygdala (green) can feed into this circuit contributing to altered sensory perception and emotional context

Central to the networks involved in psychosis and schizophrenia, the thalamus acts as a relay for most information going to the cortex^64^. Brain imaging studies have demonstrated that medication-naive patients with schizophrenia have significantly reduced thalamic and caudate volumes relative to healthy controls and medicated patients^65^. Moreover, reduced thalamic volumes has also been observed in UHR subjects^66^. A simplified schematic of the networks that may be especially relevant to psychotic symptoms in schizophrenia is presented in Fig. 2. The thalamus forms a circuit with the associative striatum and PFC whereby impairments in any of these regions can impair the functionality of the network as a whole. In addition, the hippocampus and amygdala, which are both involved in sensory perception and emotional regulation, can affect this network via their connectivity with the thalamus (but other indirect pathways also exist). Although this is an over simplification, it highlights how psychotic symptoms could arise from multiple sources of neuropathology/dysfunction or abnormal connectivity.

## Why do antipsychotics work?

This raises important questions as to how antipsychotic drugs exert their effects. In most individuals with schizophrenia, antipsychotic treatment is effective in reducing positive symptoms^67^; therefore, excessive D2 signalling in the associative striatum appears to be critical. Stimulation of D2 and D1 receptor expressing medium spiny neurons (which are largely segregated^68^) in the associative striatum feedback indirectly to the thalamus, completing a loop that allows for feedforward-based and feedback-based signalling. The basal ganglia acts as a gateway for, or mediator of, cortical inputs^69–71^ and may represent a common pathway through which psychotic symptoms present. Therefore, excessive dopamine signalling in the associative striatum may directly lead to psychotic symptoms by compromising the integration of cortical inputs. In treatment-responsive patients, antipsychotics may attenuate the expression of psychotic symptoms by normalising excessive D2 signalling^29^ to restore the balance between D1 and D2 receptor pathways^72^. Because they act downstream to schizophrenia-related presynaptic abnormalities, they fail to improve indices of cortical function (i.e., cognitive symptoms). Alternatively, impaired cortical input to the associative striatum via the thalamus, PFC or other regions could dysregulate this system independently of, or in addition to, associative striatal dopamine dysfunction. In this case, D2 receptor blockade may be insufficient to restore normal function, which is one explanation for why some individuals are treatment refractory. For example, increases in subcortical synaptic dopamine content^29^ and increases in presynaptic striatal dopamine function^52^ are both associated with increased treatment efficacy. Thus, in treatment-resistant subjects, there is little evidence of abnormal dopaminergic function^29, 52^. Medicated persons with schizophrenia, who remain symptomatic with auditory hallucinations, show increased thalamic, striatal and hippocampal activation^55^. Moreover, treatment-refractory patients who respond positively to clozapine treatment show alterations in cerebral blood flow in fronto-striato-thalamic circuitry, suggesting clozapine is restoring a functional imbalance in these systems^73^. Taken together, this evidence suggests that psychosis is the result of a network dysfunction that includes a variety of brain regions (and multiple neurotransmitter-specific pathways), of which impairment at any level could precipitate psychotic symptoms.

**Fig. 3:**
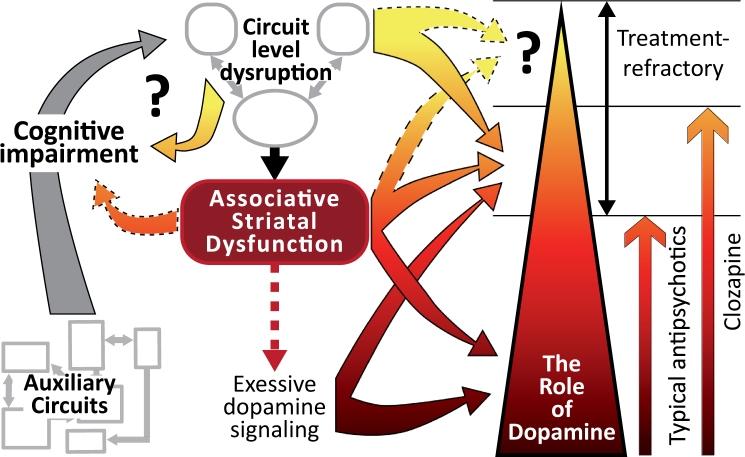
Psychosis: a consequence of severe circuit specific cognitive impairment. This schematic representation highlights the potential for cognitive symptoms to feed into psychosis networks and create positive feedback loops that spiral to psychosis. Non-specific and heterogeneous deficits in auxiliary neurocircuitry (in the context of psychosis) lead to broad cognitive impairments unique to each individual. These systems feed into the primary psychosis networks leading to destabilisation of associative striatal dysfunction and further cognitive impairment. In most individuals with schizophrenia, excessive dopamine signalling in the associative striatum leads to positive symptoms. Antipsychotics antagonise downstream D2 receptor signalling to blunt the expression of symptoms. In treatment-refractory patients (those who do not respond to first-line antipsychotics) blocking D2 receptors is insufficient to blunt positive symptoms suggesting further upstream dysfunction in the associative striatum or psychosis networks. Clozapine may lead to improvement in some of these individuals by stabilising function throughout these networks in addition to D2 receptor antagonism. Positive symptoms in treatment-refractory patients who fail to respond to clozapine may be the result of severe impairment throughout psychosis networks (and the associative striatum) that are independent of dopamine dysfunction. Thus, our current treatments for positive symptoms act downstream of the source of cognitive impairments, hence their ineffectiveness in treating cognitive symptoms. While the expression of psychotic symptoms may be a discrete outcome, separate to impairments in cognitive function, the upstream cause of these symptoms may share common neuropathology

Although increased positive symptom severity has been associated with impaired cognitive flexibility^74^, there is a little evidence for subcortical hyperdopaminergia playing a direct role in the cognitive impairments observed in schizophrenia. Furthermore, antipsychotic treatments do not improve patient’s cognitive function^75^. There is a mounting evidence that cognitive symptoms may present prior to positive symptoms in schizophrenia^76^. Given brain networks involved in hallucinations and delusions all involve cortical regions, the underlying pathology causing cognitive symptoms may also contribute to psychotic symptoms. Thus, in some cases psychosis may represent the summation of broad cognitive impairments inducing local network dysfunction (Fig. 3). Regardless, positive symptoms are relatively distinct in the clinical setting but the presence and severity of symptoms are determined interactively with interviews and questionnaires. The inability to do the same in other species means the best avenue for assessing animal models may be to identify outcomes that are sensitive to the underlying neurobiology observed in schizophrenia and psychosis. Given the action/effectiveness of antipsychotics, the primary downstream region of interest, in the context of elevated dopamine transmission, is the associative striatum.

## Modelling psychosis: the use of animal models

Potentially, the most useful avenue for animal models to assist in schizophrenia research will be identifying convergent aetiological pathways^77^. Understanding which neurotransmitter systems and brain regions are most involved may help to identify the core neurobiological features of schizophrenia. For example, changes in dopaminergic systems are observed in animal models after manipulation of factors based on schizophrenia epidemiology^50, 51^, genetics^78^, pharmacology^79^ and related hypotheses^80^. These include changes in early dopamine specification factors^50, 51^, sensitivities to psychostimulants^50,51,78, 80^ and alterations in dopamine neurochemistry^50,51,78, 79^. Evidence of subcortical dopaminergic hyperactivity or sensitivity in animal models is proposed to represent the face validity (i.e., mimicking the phenomenology of schizophrenia) for psychosis in patients. The most commonly used behavioural assessments of positive symptoms in animal models include enhanced amphetamine-induced locomotion and deficits in prepulse inhibition (PPI)^81^. These tests are widely used because they are relatively simple to perform. However, we propose that given current knowledge of the neurobiology in schizophrenia, they have outlived their usefulness as measures of positive symptoms.

### Amphetamine-induced locomotion

Amphetamine increases dopamine release in striatal brain regions of both humans^38^ and rats^82^. Amphetamine-related behaviours in rodents are also strongly linked to activity in striatal brain regions^82, 83^. Thus, an increased locomotor response to amphetamine (and other psychostimulants, which face similar criticisms) is considered a simple test to reflect the subcortical hyperdopaminergia underlying the psychotic symptoms in schizophrenia. Most animal models of schizophrenia report increased locomotor activation after psychostimulants^78^. However, the recent clinical evidence described above suggests that current assessments of animal models does not reflect contemporaneous knowledge of dopamine activity in those with schizophrenia.

The relative contribution of specific dopamine pathways to amphetamine-induced locomotion provides a good example of why a paradigm shift is required for research using animal models for positive symptoms in schizophrenia. For example, amphetamine-induced locomotion is largely driven by limbic dopamine release. Local administration of amphetamine^84–87^ or dopamine^84,88, 89^ into the nucleus accumbens induces locomotion. Furthermore, blocking dopamine signalling in the nucleus accumbens attenuates amphetamine-induced locomotion^90^. Specifically activating limbic dopamine projections using chemogenetic tools robustly increases locomotion, but activating associative dopamine projections does not^91^. Thus, there is an anatomical misalignment between the primary behavioural outcome deemed important for positive symptoms in animal models of schizophrenia (i.e, psychostimulant-induced locomotion driven by limbic dopamine), and clinical evidence in patients (hyperactive associative striatal dopamine). Furthermore, clinical studies directly comparing activity levels in patients with schizophrenia and bipolar disorder suggest that hyperactivity may be a core feature of bipolar disorder rather than schizophrenia^92^.

One argument for amphetamine-induced locomotion is that it is predictive of antipsychotic efficacy, but this is merely a serendipitous side effect. Systemically administered amphetamine increases dopamine function in both the limbic striatum (locomotion) and associative striatum (positive symptoms). Systemically administered antipsychotics antagonise D2 receptors throughout the brain. Therefore, amphetamine-induced locomotion acts serendipitously to predict antipsychotic effectiveness via dopamine release in a parallel circuit (limbic vs. associative dopamine). Optimally, antipsychotics that diminish dopamine signalling preferentially in the associative, rather than the limbic, striatum need to be developed. Obviously, amphetamine-induced locomotion would not be predictive for the latter treatment options.

### Prepulse Inhibition

One of the most consistently observed neurological impairments in schizophrenia is impaired sensorimotor gating in the form of decreased PPI^93, 94^. Deficits in PPI may reflect an inability to gate out irrelevant information. PPI deficits also respond to antipsychotics but are not specific to schizophrenia^93, 94^. Thus, PPI deficits do not represent a specific or diagnostic trait of schizophrenia. Intact cortical and striatal function are critical for PPI^95^ and, therefore, deficits in PPI also reflect an interface between positive and cognitive symptom groups^81^.

PPI is assessed almost identically in rodents and humans and, therefore, is one of the most widely studied deficits in schizophrenia. In rodents, the contribution of limbic dopamine projections to PPI are well-known^95^, though the associative striatum has also been implicated^96, 97^. Thus, PPI deficits clearly lack specificity concerning the hyperdopaminergia observed in schizophrenia. Therefore, when assessing rodent models, PPI impairments alone are insufficient for determining positive symptom phenotypes and their predictive validity suffers from the same criticism as that of amphetamine-induced locomotion (parallel blockade of limbic D2 receptors).

## Can we objectively test positive symptom connectivity in rodents?

**Fig. 4:**
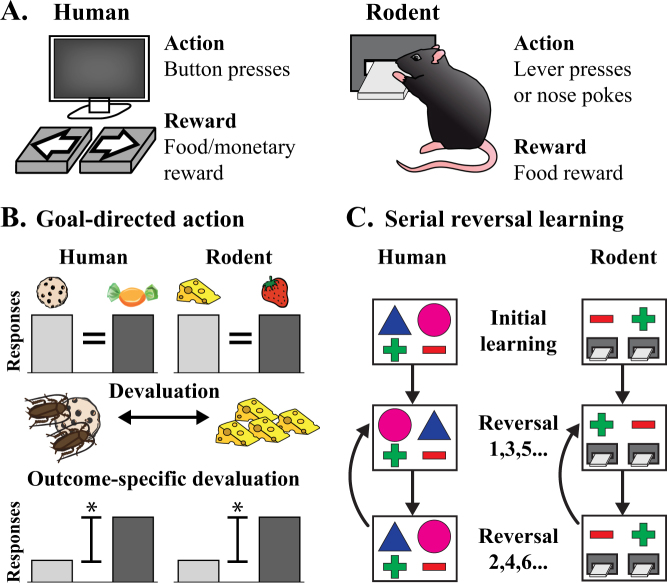
Comparisons for cognitive tests in humans and rodents. Humans and rodents can both perform cognitive tasks that feature actions to obtain rewards **(a)** The primary differences in testing are that humans can receive monetary rewards whereas rodents tend to be given food rewards. Furthermore, rodents require more initial training to learn the action (i.e., lever pressing or nose poking). To test for goal-directed action **(b)** both humans and rodents are trained to associate two actions (left and right button/lever presses) with two separate food rewards. One of these rewards is then devalued through an aversive video (cockroaches on the food item) for humans or feeding to satiety in rodents. Healthy controls will demonstrate outcome-specific devaluation by biasing their response towards the food reward that was not devalued. Serial reversal learning **(c)** requires the subject to learn a simple discrimination between two choices of which one is associated with a reward. Once certain criteria are met, the contingencies are reversed so that the non-rewarded stimulus is now rewarded and the previously rewarded stimulus does not attain a reward. This is classified as the first reversal. Once the criteria are met for the new contingencies, the rewarding stimulus is switched again (back to the original pairings) for the second reversal. This switching back and forth continues until completion of the test

Clearly, alternative behavioural phenotypes in animal models, consistent with the underlying neuroanatomical/biological features of schizophrenia, need to be established. This does not invalidate our current rodent models; it just emphasises that, in light of the recent compelling PET evidence in patients, we need to review their relevance to the positive symptoms of schizophrenia. Psychosis, an extremely ‘human’ syndrome, will never be truly observable in rodents. However, we can, and should, aim to establish more translationally relevant tests for the underlying neurobiology of psychosis. Ultimately, we need better behavioural tests for positive symptoms in animal models that will lead to therapies efficacious for both positive and cognitive symptoms in patients. We contend that tests aimed at understanding associative striatal function are imperative. We propose that a combination of cognitive behavioural tasks, that can be tested similarly in humans and rodents (Fig. 4a), represents our best opportunity to assess positive symptom neurobiology in animal models. It is important to consider that neither task alone is a reliable indicator of positive symptom neurobiology (as these tasks assess cognitive function and outcomes are therefore relevant to cognitive symptoms); however, in combination they can help isolate associative striatal function.

### Goal-directed action: sensitivity to outcome devaluation

**Fig. 5:**
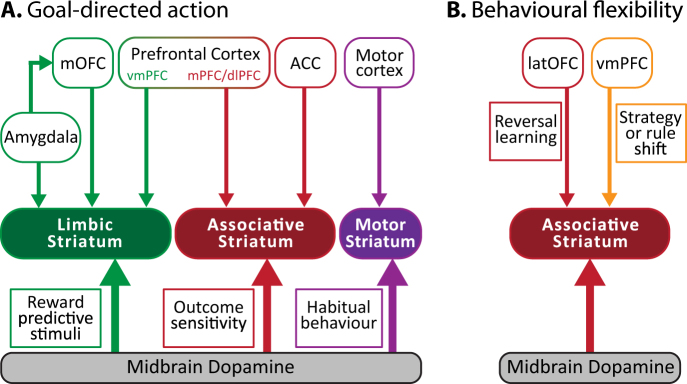
Behavioural tests to probe associative striatal function. **a** The neurocircuitry involved in goal-directed action can be split into three primary circuits. The associative system (red), including the PFC and ACC, is required for the acquisition and expression of goal-directed action, which is sensitive to outcome devaluation. In contrast, the limbic system (green) is critical for the formation of associations between reward predictive stimuli and action. Habitual behaviours rely on the sensorimotor system (purple). **b** Behavioural flexibility involves OFC and PFC inputs to the associative striatum. The OFC is critical for reversal learning whereas the PFC is required when shifting to new rules or strategies. The associative striatum is the only common region required for goal-directed action that is sensitive to outcome devaluation and serial reversal learning. OFC orbitofrontal cortex, PFC prefrontal cortex, ACC anterior cingulate cortex, vm ventromedial, m medial, dl dorsolateral, lat lateral

Goal-directed behaviour is critical for understanding the relationship between actions and their consequences in both humans and rodents. Moreover, goal-directed action heavily depends on the function of the associative striatum^21,24, 98–100^ and can be assessed using near identical behavioural paradigms in both humans^101^ and rodents^102^ (Fig. 4b). To test impairments in the learning of action-outcome associations in humans and rodents the sensitivity to outcome-specific devaluation can be determined. Outcome-specific devaluation is useful way of establishing that an action is goal-directed and that the correct action–outcome associations have been formed. In order to test this, after training to associate actions with specific outcomes (action–outcome association), one of the outcomes is devalued. After devaluation, when the subject is given the choice between the two action-outcome pairs, healthy controls respond more for the outcome that was not devalued. This demonstrates the ability to establish action-outcome associations correctly and adapt actions based on newly acquired information. The specific neurocircuitry involved in goal-directed behaviour is based on years of associative learning research^103^. Sensitivity to outcome devaluation is dependent on the PFC and associative striatum (Fig. 5a). Impairments in goal-directed action in schizophrenia have been associated with altered caudate function^101^ and disorganised thought^104^. Importantly, the insensitivity to outcome devaluation observed in persons with schizophrenia was not due to impairments in reward sensitivity after devaluation (i.e., limbic systems) but rather, reflected an inability to use this information to direct choice^101^.

### Behavioural flexibility: serial reversal learning

One limitation of outcome-specific devaluation is that it does not allow for the delineation of functional deficits in the PFC vs. associative striatum. Thus, pairing this task with another that relies on the associative striatum, but not the PFC, is required. The basal ganglia is also involved in flexible decision-making and specifically reversal learning (the ability to adapt when outcome contingencies are reversed) which can be tested similarly in humans and rodents (Fig. 4c). Extensive work in rodents, primates and humans have demonstrated that specific forms of behavioural flexibility are dependent on differing neurocircuitry^69,105, 106^(Fig. 5b). For example, the orbitofrontal cortex and associative striatum are critical for reversal learning when re-exposed to previous contingencies (e.g., serial reversal learning). In contrast, the PFC is critical for shifting from one rule or strategy to another (i.e., attentional set-shifting). Thus, deficits in serial reversal learning are particularly sensitive to orbitofrontal cortex and associative striatal dysfunction but not PFC dysfunction. Persons with schizophrenia exhibit deficits in both attentional set-shifting and reversal learning^107^. Deficits in reversal learning are independent of working memory deficits and have also been associated with disorganised thought^108^.

### A circuit level approach to positive symptoms in animal models

Advances in behavioural neuroscience have helped to delineate specific circuits important for aspects of complex behaviour. Moreover, improvements in circuit isolation using techniques such as optogenetics^109^ or chemogenetics^110^ mean the field is at a point now where we can focus on particular brain regions and circuits. The proposed tasks, outcome-specific devaluation and serial reversal learning, provide a potential mechanism to focus on associative striatal function (Fig. 5). For example, an insensitivity to outcome devaluation and impaired serial reversal learning would be predicted if associative striatal function is compromised. In contrast, an insensitivity to outcome devaluation but maintained serial reversal learning would predict impairments in PFC function. The opposite would be true if orbitofrontal cortex dysfunction is present. Although this is far from perfect, an understanding of the extended connectivity from the associative striatum provides a starting point to probe animal models of schizophrenia to determine whether they demonstrate true associative striatal dysfunction rather than limbic dysfunction. Like psychosis, deficits in goal-directed behaviour^103^ and reversal learning^107^ are observed in a multitude of disorders other than schizophrenia, meaning that multiple tests assessing cognitive function and other circuitry will still be required to determine how useful one particular animal model will be to an individual psychiatric condition. This combination of tests, however, will allow for a more selective assessment of associative striatal function.

## Challenging longstanding assumptions and moving forward

Clozapine, discovered in the 1960’s, remains the most effective antipsychotic medication, although its use is restricted due to its side effect profile^111^. This stagnation in drug development for schizophrenia highlights a key weakness in schizophrenia research; a lack of effective bi-directional translation between basic and clinical research. The fact that the current methods of testing for psychotic symptoms in rodents are now misaligned with recent clinical evidence indicates a need to advance how positive symptoms are examined in animal models. We have proposed a combination of behavioural tests in rodents that are sensitive to dysfunction at the primary site of dopaminergic neurobiology observed in schizophrenia. There will never be a perfect model for psychosis in rodents, but it is critical that we acknowledge the limitations of current methods so that an active dialogue is established.

It is also imperative that basic and clinical researchers maintain active collaborations to prevent the misinterpretation or mistranslation of animal studies. For example, based on the work in monkeys and rodents^112–114^, the results of D1 receptor agonists on working memory function in schizophrenia have been largely negative^115–117^. One contributing factor may have been that the preclinical studies tested delay-dependent working memory (i.e., how long a piece of information is kept in working memory), whereas the clinical studies tested a differing working memory construct, memory span capacity (i.e., how many items can be kept in working memory at one time). Other factors such as medication history^118^ may also interfere with the effectiveness of translation between preclinical and clinical studies. To improve translational schizophrenia research it is imperative that we build better avenues for communication between basic and clinical research teams to avoid the aforementioned issues.

## Conclusion

Complex syndromes like schizophrenia require a constant reformulation and evolution of ideas and strategies that cannot be achieved by either basic or clinical research in isolation. Clinically, the point at which psychotic symptoms become apparent has dictated our primary diagnostic criteria. Furthermore, it has become evident that a range of complex symptoms emerge before this diagnostic time point. Clinical research must continue to elucidate the features associated with the development of psychosis and better inform a patient’s clinical trajectory throughout the course of schizophrenia. However, our current ability to model psychotic symptoms in animal models is at best questionable and based on historical presumptions rather than recent clinical evidence. Thus, it is imperative that basic research using animal models develops objective measures for the neurobiology underlying psychosis in schizophrenia. Understanding in detail the neurobiological processes that precede these behavioural abnormalities, an avenue of research that cannot be conducted in humans, now becomes a priority. It is only by a synthesis of such approaches that novel therapeutic targets and treatments will emerge.
